# Multimodality Surgical Approach in Management of Laryngotracheal Stenosis

**DOI:** 10.1155/2018/4583726

**Published:** 2018-04-01

**Authors:** Ashfaque Ansari, Annju Thomas

**Affiliations:** E. N. T. Department, MGM Medical College and Hospital, Aurangabad, Maharashtra, India

## Abstract

**Introduction:**

Postintubation laryngotracheal stenosis requires a precise diagnosis and an experienced operator in both endoscopic and surgical treatment. This report presents surgically treated cases of laryngotracheal stenosis secondary to long-term intubation/tracheostomy with review of the literature.

**Materials and Methods:**

In this retrospective study, we present 5 cases (a 23-year-old male, 13-year-old male, 22-year-old male, 19-year-old male, and 33-year-old female) of postintubation/tracheostomy laryngotracheal (glottic/subglottic) stenosis in the years 2016 and 2017. Each patient was managed differently. Intubation characteristics, localization of stenosis, surgical technique and material, postoperative complications, and survival of patients were recorded.

**Results:**

The site of stenosis was in the subglottis in 4 patients and glottis in 1 patient. The mean length of the stenosis was greater in the postintubation group. Postintubation stenosis had a mean duration of intubation of 6.8 days, compared to 206.25 days of cannulation following tracheostomies. Each patient underwent an average of 2 procedures during their treatment course. One patient underwent open surgical anastomosis because of recurrent subglottic stenosis after multiple treatments. Phonation improved immediately in almost all except in the patient who underwent only endoscopic dilatation.

**Discussion:**

The reasons for laryngeal stenosis and its delayed diagnosis have been reviewed from the literature. Suture tension should be appropriate, and placement of the suture knot outside the trachea minimizes formation of granulation tissue. The published reports suggest that resection by endoscopy with laser and open technique resection and primary anastomosis are the best treatment modality so far as the long-term results are concerned.

**Conclusion:**

Resection of stenotic segment by open surgical anastomosis and laser-assisted resection is a safe option for the treatment of subglottic stenosis following intubation without the need for repeated dilation. Endoscopic dilation can be reserved for unfit patients.

## 1. Introduction

Laryngotracheal stenosis is a congenital or acquired narrowing of the airway that may affect the supraglottis, glottis, and/or subglottis. It has several grades. Incidence of laryngotracheal stenosis following intubation has been reported up to 21% [1]. However, only a few (1-2%) of these patients present with the symptoms. Resection and anastomosis has been established as the definitive treatment of stenosis more than one cm in length [2]. The current study aims to present cases of laryngotracheal stenosis managed by different methods at our hospital.

## 2. Materials and Methods

### 2.1. Setting and Patients

The study was conducted by the department of ENT in a teaching hospital in western India. Patients were identified from the prospectively maintained admission and operation theater registers. A total of 5 patients were identified between April 2016 and November 2017. The 5 patients were treated for glottic/subglottic stenosis following tracheostomy and after prolonged endotracheal intubation. Cases with high-grade stenosis (Cotton–Meyer's III and IV) with loss of structural integrity were included in this study. Patients having GERD were excluded.

Demographic data were obtained for each patient: age, sex, comorbid conditions, and corticosteroid therapy received. The following data regarding the stenosis were also obtained: the circumstances leading to the development of stenosis (postintubation/tracheostomy/surgery/trauma), type of stenosis (web-like stenosis, granulation tissue formation, and adhesions), and the procedures performed.

Each patient underwent a standard preoperative assessment, including physical examination, routine laboratory tests, chest radiography, and computed tomography of the chest with virtual bronchoscopy. An initial diagnostic flexible bronchoscopy was performed for each patient to identify the type, location, and severity of the stenosis. The stenosis was characterized severe if it was causing symptoms, primarily dyspnea, and was complex in nature (stenosis combined with cartilage fracture or tracheomalacia), and the obstruction of the tracheal lumen exceeded 50%.

### 2.2. Case Report 1

A 23-year-old male came to our OPD with complaints of change of voice and noisy breathing. Examination revealed that the young man had inspiratory stridor and hoarseness of voice with portex tracheostomy tube in situ. History revealed that he was admitted previously for organophosphorus poisoning 3 years back, during that time he was intubated for 15 days. After extubation, he was kept in the ward for 5 days after which he was discharged. The patient developed dyspnea 1 month after discharge from primary hospital, and the patient was admitted and taken up for emergency tracheostomy, following which a clinical diagnosis of tracheal stenosis was made.

CT neck revealed tracheal stenosis proximal to tracheostomy stoma involving length of 2 cm and transverse diameter of 6 mm.

After which, the patient underwent 6 check bronchoscopies and 1 dilatation and followed stenting of the stenosis at the previous hospital, which did not relieve him of his complaints. He underwent a check bronchoscopy with dilatation of the tracheal stenosis at our hospital 1 year later.

**Figure 1:**
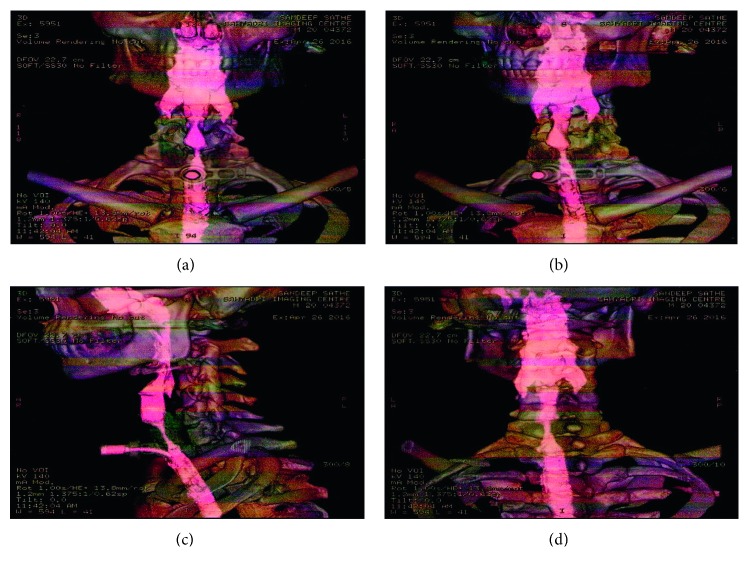
3D CT neck showing stenotic portion.

**Figure 2:**
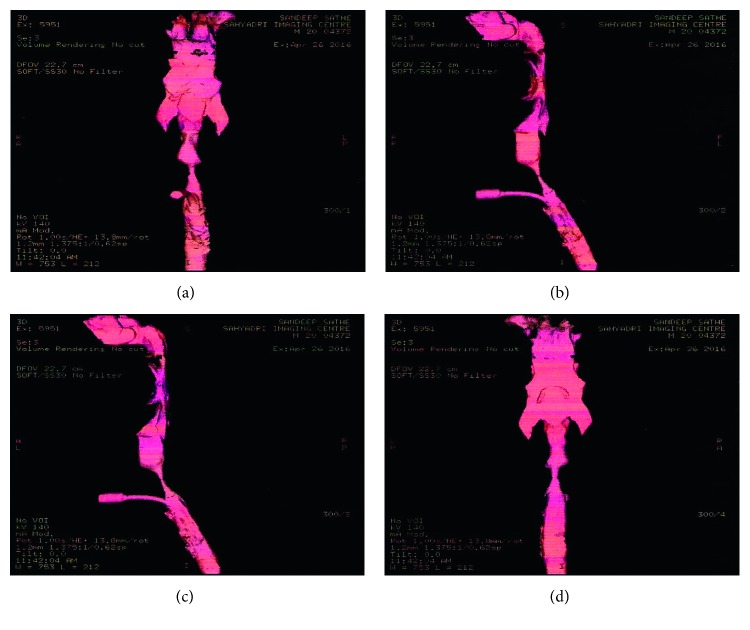
3D CT neck showing stenotic portion.

The patient returned back to our ENT OPD 1 year after the dilatation with complaints of hoarseness of voice and stridor. 3D CT neck (Figures 1 and 2) revealed tracheal stenosis over approximately 11 mm length, noted at the level of cricoid cartilage. Transverse diameter measures 5.5 mm, and AP diameter measures 5.1 mm. There was grade III (90%) stenosis over the third tracheal ring with narrowing extending proximally up to the second ring. CT scan showed a stenotic segment of 2.5 cm. The patient and his relatives were explained about the condition and the procedure to be performed. They agreed for a reconstructive surgery, and a tracheal resection and anastomosis was planned.

The patient underwent surgery, and apron's neck incision was taken. Flaps elevated superiorly till the hyoid bone and inferiorly till the clavicle. Strap muscles were separated, and thyroid gland was dissected. Trachea was visualized, and stenotic portion of the trachea was resected, and end-to-end anastomosis was done with 3/0 Prolene suture material, and a Romo vac drain was kept in situ. A mentosternal suture with neck in flexion to restrict neck movement was placed with 1/0 Prolene. Postoperatively, the patient was kept in ICU with nasal intubation. Nasal intubation and Romo vac drain was removed on the 5th postoperative day. The patient was shifted out of ICU on the 8th postoperative day due to uneventful recovery and was discharged on the 14th postoperative day. At 1-month follow-up, the patient was asymptomatic with normal breathing and voice.

**Figure 3:**
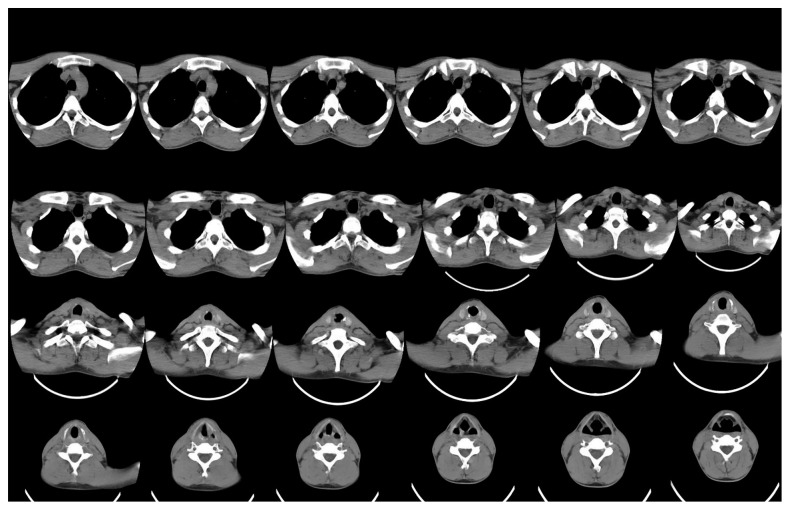
CT neck 6 month postoperatively.

CT neck 6 month postoperatively (Figure 3) suggested of no evidence of stenosis. Tracheal lumen and tracheal bifurcation were normal, and on 70 degree scopy, vocal cord mobility was normal.

### 2.3. Case Report 2

A 13-year-old male was referred to our OPD with complaints of dyspnea and noisy breathing. Examination revealed that the patient had inspiratory stridor with metallic tracheostomy tube in situ. On indirect laryngoscopy, bilateral vocal cords were mobile. History revealed that the patient was admitted in the Government Medical College in view of neuroparalysis secondary to snake bite. The patient was ventilated through an endotracheal tube for 10 days after which he underwent a tracheostomy. The patient was shifted to the ward on the 23rd day after admission. The patient was discharged with a metallic tube in situ. After discharge, the patient complained of noisy breathing. The patient underwent flexible bronchoscopy which revealed subglottic stenosis, bilateral fixed vocal cords due to thickening, and fibrosis. The patient was referred to our hospital for further management. The patient's relatives were explained about the condition and the procedure to be performed. They agreed for the surgery, and laser-assisted transglottic stenosis excision was planned.

**Figure 4:**
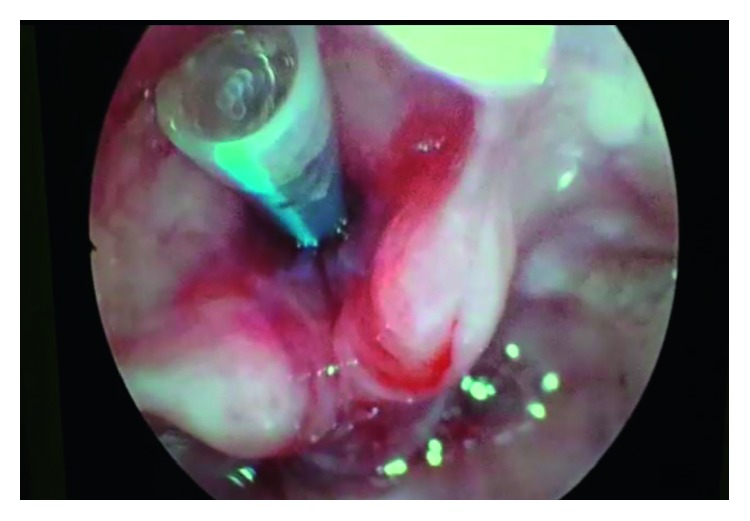
Endotracheal tube number 4 inserted after laser-assisted release of stenosis.

**Figure 5:**
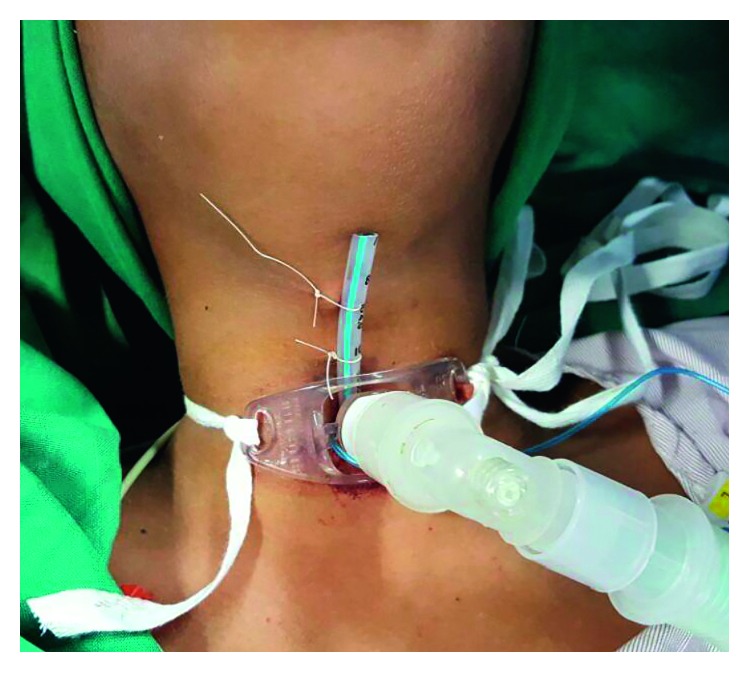
Endotracheal tube number 4 inserted after laser-assisted release of stenosis.

The patient underwent the surgery under general anaesthesia, and flexible bronchoscopy was done. Floppy epiglottis was visualized, and glottis stenosis with interarytenoid adhesion was seen. Subglottic soft stenosis (Cotton–Meyer's grade 4) was visualized. Rigid bronchoscopy was done, and stenotic segment length was assessed. Bogdasarian grade 4 postglottic stenosis was noted and partly released with CO_2_ laser. Bougie was used for dilatation of the subglottic stenosis. CO_2_ laser was used to release the stenotic bands of the subglottic stenosis. Endotracheal tube number 4 was inserted through the tracheostomy (Figures 4 and 5) site upward in a retrograde fashion through the stenosis to maintain patency.

Postoperatively, the patient was kept in ICU. The patient was shifted out of ICU on the 3rd postoperative day. The patient made uneventful recovery with no complaints of dyspnea and noisy breathing. The patient was discharged on the 20th postoperative day after Montgomery T-tube insertion. Postoperatively, after the 6 month, the Montgomery T-tube was removed. One month after T-tube removal, there was no evidence of stridor and no recurrence of stenosis.

### 2.4. Case Report 3

**Figure 6:**
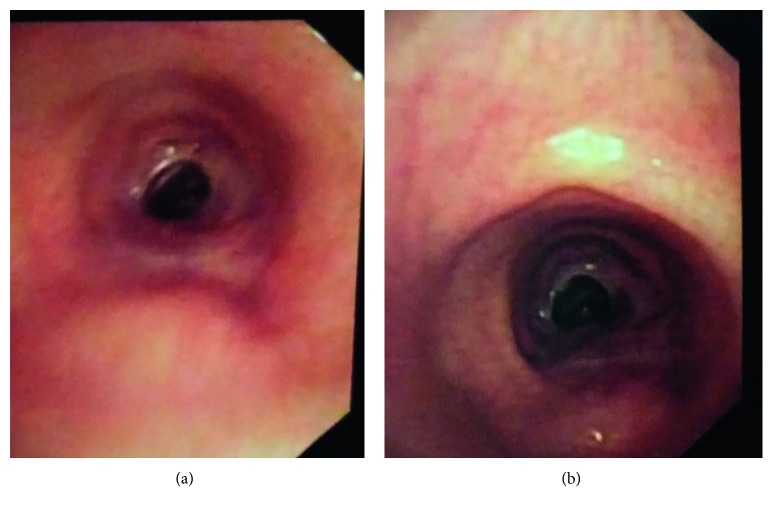
Stenosis seen 4 cm below the glottis.

A 22-year-old male presented to our OPD with complaints of dyspnea on lying down and hoarseness of voice. Examination revealed that the patient had orthopnea and stridor. On indirect laryngoscopy, bilateral vocal cords were fixed with interarytenoid adhesion and with subglottic stenosis. History revealed that the patient had history of hospitalization in view of OP poisoning. The patient was intubated for 2 weeks. The patient was shifted to the ward after extubation on the 3rd week after admission. The patient was discharged. After discharge, the patient complained of hoarseness of voice and dyspnea. The patient came to our OPD where on indirect laryngoscopy it was revealed that the patient has bilateral abductor cord palsy with interarytenoid adhesion, and on flexible bronchoscopy, there was a stenosis seen, 4 cm (Figure 6) below the glottis which was grade II soft stenosis. The patient's relatives were explained about the condition of the patient and the procedure to be performed. They agreed for the surgery, and MLscopic Kashima's cordotomy by laser was planned.

**Figure 7:**
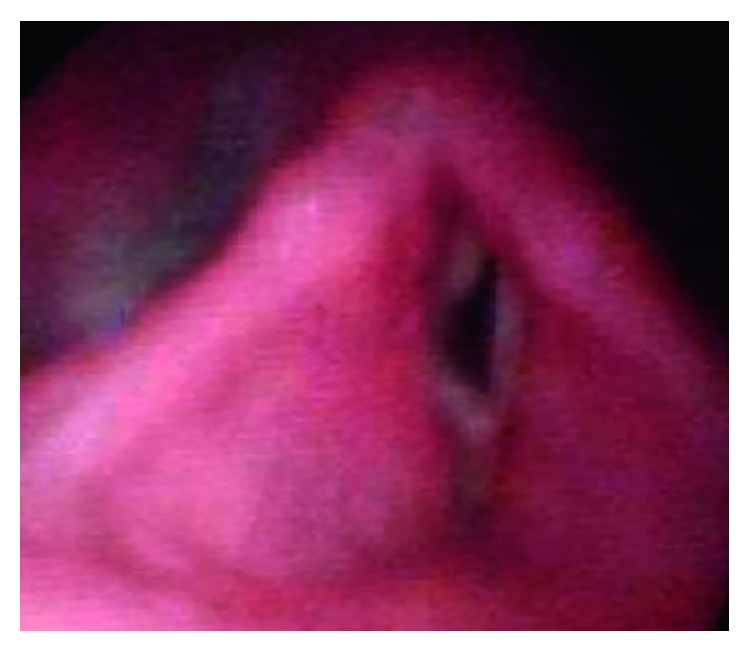
Postoperative image of Kashima's cordotomy.

The patient underwent surgery on 18 March 2017. Under general anaesthesia, after intubation, MLscope was introduced. Bilateral vocal cords were visualized, and posterior commissure was visualized. Right side cordotomy was done using CO_2_ laser (Figure 7), followed by dilatation of stenotic portion using rigid bronchoscope. The patient was shifted to ICU postoperatively and was shifted out on the 3rd postoperative day. The patient made an uneventful recovery and was discharged from the ENT ward on the 9th postoperative day. Six months postoperatively, on endoscopic examination, there was no evidence of glottic or subglottic stenosis and there was no evidence of stridor.

### 2.5. Case Report 4

A 19-year-old boy was brought to our OPD with complaints of dyspnea. Examination revealed that the patient had stridor and portex tracheostomy tube in situ. On indirect laryngoscopy, bilateral vocal cords were mobile. History revealed that the patient was admitted in another hospital 1 month prior to visit at our hospital in view of head injury. The patient had undergone craniotomy for the same with tracheostomy. The patient was discharged after an uneventful recovery after decannulating him. The patient started developing dyspnea 15 days after discharge. The patient underwent tracheostomy once again. The patient was discharged with portex tracheostomy tube number 8. The patient came to our OPD in view of dyspnea after which a CT neck was performed suggestive of tracheostomy in situ 3 cm from the carina with tracheal stenosis at the level of tracheostomy tube. The patient's relatives were explained about the condition of the patient and the procedure to be performed. They agreed for the surgery, and flexible bronchoscopy was planned.

**Figure 8:**
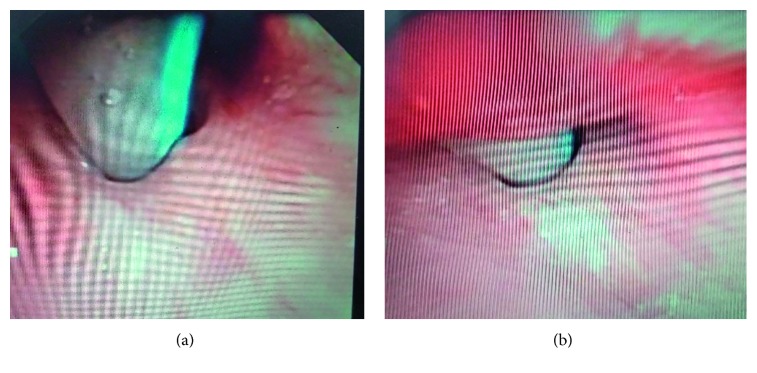
Tracheal stenosis at level of tracheostoma.

The patient underwent surgery on 20 September 2016. Under local anaesthesia, flexible bronchoscopy was done. Portex tracheostomy tube no. 8 was slightly pulled out for better visualization. Tracheal stenosis was visualized at level of tracheostoma (Figure 8). Tracheal stenosis was mild, and granulation tissue was positive. Dilatation was done using balloon dilator, and tracheostomy tube number 7 was inserted. The patient was shifted to the ward postoperatively and was discharged with metallic tracheostomy tube on the 3rd postoperative day. The patient followed up on the 15th postoperative day, and tracheostomy decannulation was done. After check bronchoscopy and dilatation, the patient was discharged. The patient required one more sitting of dilatation in the 2nd postoperative month. On the 6th postoperative month, check bronchoscopy revealed no evidence of stenosis.

### 2.6. Case Report 5

A 33-year-old female was referred to our OPD with complaints of hoarseness of voice and dyspnea on exertion. Examination revealed that the patient had stridor. On indirect laryngoscopy, bilateral vocal cords were mobile. History revealed that the patient was admitted in view of clozapine poisoning and was kept intubated for 10 days. After extubation, the patient made an uneventful recovery and was discharged on the 5th day after shifting to the ward. The patient came to our OPD with complaints of hoarseness of voice and dyspnea on exertion. CT scan revealed narrowing of trachea for an approximate length 2 cm from C7-T1 to T1-T2. Since the stenosis involved the distal airway fibreoptic visualization with holmium laser excision was planned. The patient's relatives were explained about the condition of the patient and the procedure to be performed.

**Figure 9:**
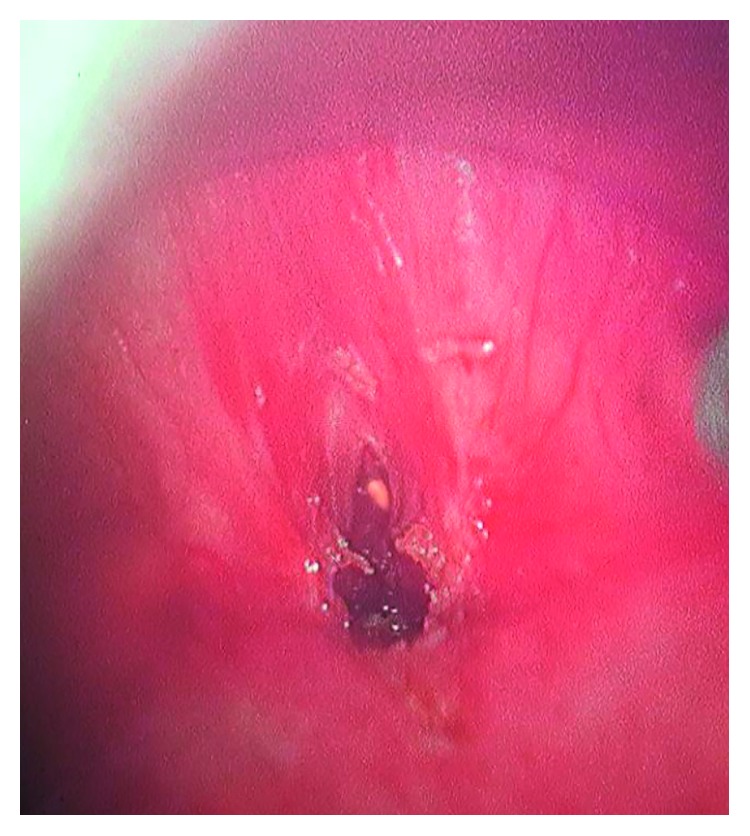
Laser excision of stenosis.

They agreed for the surgery and excision of stenosis by holmium laser (Figure 9) with balloon dilatation followed by mitomycin C application was done. The patient was shifted to ICU postoperatively and was shifted out on the 2nd postoperative day. The patient made an uneventful recovery and was discharged from the ENT ward on the 5th postoperative day. There was no evidence of stridor and no evidence of stenosis on check bronchoscopy when the patient followed up in the 4th postoperative month.

## 3. Results

**Figure 10:**
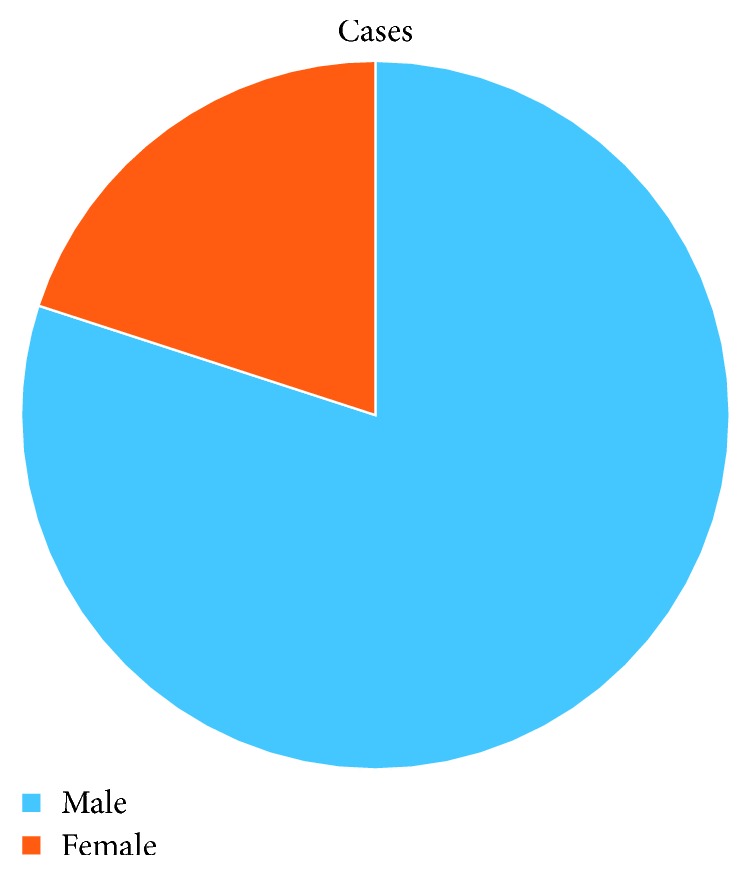
Male and female pie graph.

Five patients were included: 1 female and 4 males (Figure 10). Patients were predominantly male in the case study. Mean age was 22 ± 10 years (range: 13 to 33 years). Reason for intubation/tracheostomy is noted in Table 1.

The comorbidities found were diabetes mellitus and hypothyroidism (1 patient each). Two each were addicted to smoking and alcohol. Three patients were on corticosteroid therapy.

**Figure 11:**
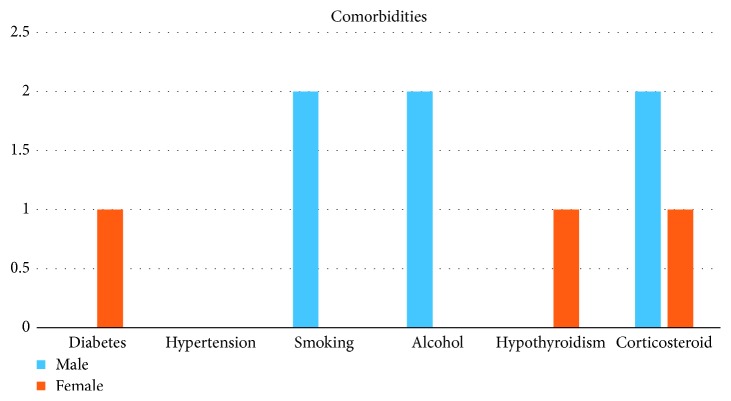
Comorbidities in males and females.

Patients' characteristics and comorbidities as seen in patient at the time of the first interventional bronchoscopy procedure performed are listed in Table 2 and Figure 11.

**Figure 12:**
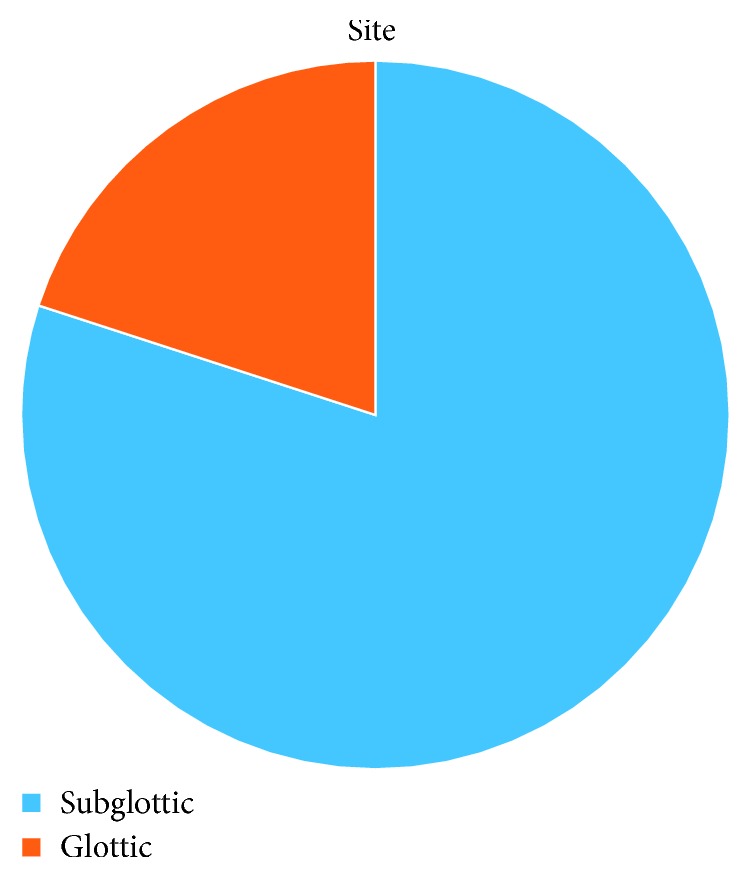
Site of stenosis.

The site of stenosis was localized in the subglottis in 4 patients (80%) and glottis with subglottis in 1 patient (20%) (Figure 12).

The characteristics of the laryngotracheal stenosis varied depending on whether the development of stenosis followed postintubation or tracheostomy and are presented in Table 3. The mean length of the stenosis was greater in the postintubation group due to the formation of a web-like stenosis along the distribution of the endotracheal tube cuff. Patients with postintubation stenosis had a mean duration of intubation of 6.8 days. Patients with tracheal stenosis following tracheostomy had a much longer duration of cannulation, with a mean of 206.25 days.

**Figure 13:**
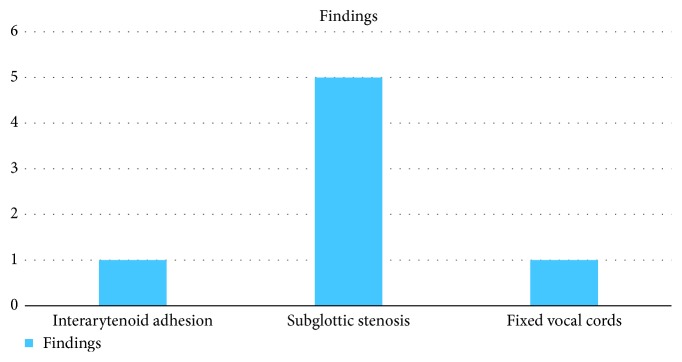
Findings/type of stenosis.

The types of the glottic stenosis developed in the patients are presented in Figure 13. No significant difference was noted between males and females with regards to the predisposing factors or comorbid conditions and the type of glottic stenosis. A variety of modalities and treatments were applied as mentioned in Table 4.

**Figure 14:**
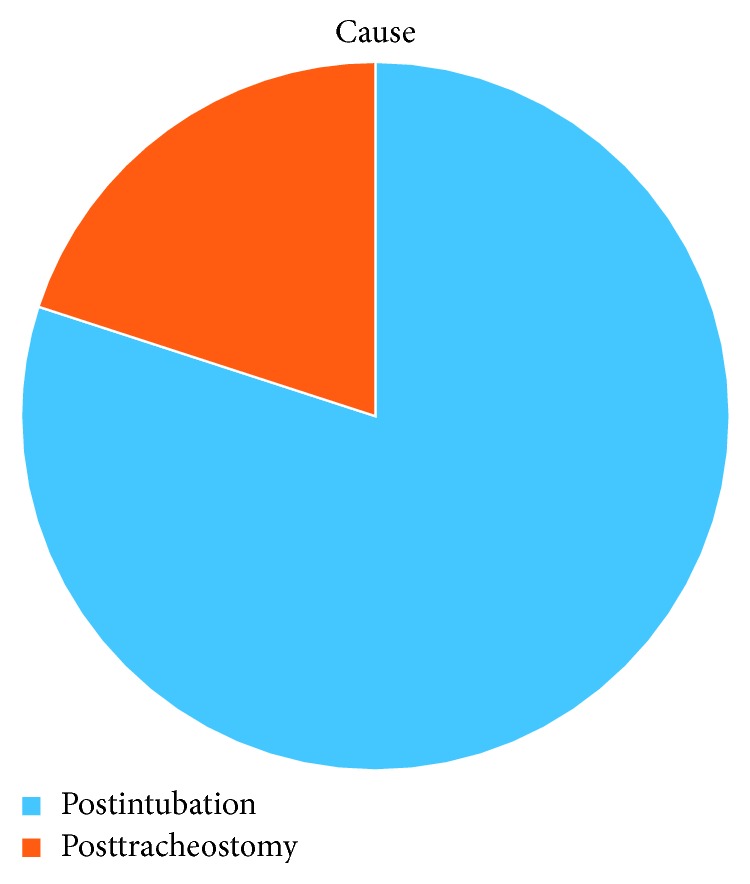
Main cause of trauma.

All patients underwent indirect laryngoscopy upon admission. No complications related to the procedure were reported. Four patients underwent flexible bronchoscopy first. HCT3D was performed in 1 patient. Postintubation injury was the most frequent cause of stenosis in 4 patients (80%) and 1 due to posttracheostomy trauma (20%) (Figure 14).

Each patient underwent an average of 2 procedures during their treatment course. One patient was treated with 5 procedures prior to admission and finally underwent surgical management by anastomosis. This patient underwent open surgery because of recurrent subglottic stenosis after multiple treatments with rigid bronchoscopy and stent implantations over a three-year period. Three out of 5 patients were admitted with an in situ tracheostomy tube. Postoperatively, 1 patient out of 5 was discharged with a stent.

### 3.1. Preoperative Characteristics

The median time of intubation causing tracheal stenosis was 6.8 [7–10] days All the patients had complex lesions (>1cm). Two patients had grade 3 subglottic stenosis (40%), two patients had grade 2 subglottic stenosis (40%), and one patient (20%) had bilateral vocal cord fixation with interarytenoid adhesion and subglottic stenosis. Of the five patients, two patients had previous endoscopic dilation, and one of the 2 patients who had underwent dilatation in the past had tracheal stents prior to surgery.

### 3.2. Operative Findings

An endoscopic visualization of the glottis and subglottis was sufficient in 4 patients of which 1 (20%) patient with bilateral vocal cord paralysis underwent endoscopic laser-assisted cordotomy with removal of interarytenoid adhesion and subglottic stenosis, 1 (20%) patient with subglottic stenosis with lax tracheal underwent endoscopic laser-assisted resection with placement of stent, 1 (20%) patient with stenosis at the level of tracheostoma underwent dilation using bougies, and one patient (20%) with subglottic stenosis at the level of C7-T1 and T1-T2 underwent excision of stenosis by holmium laser followed by dilatation of excised stenotic part by balloon followed by mitomycin C application around the excised region. Only one patient of the five (20%) with subglottic stenosis with a history of previous endoscopic dilations and stenting which were unsuccessful underwent open surgical tracheal resection and anastomosis.

### 3.3. Postoperative Results

**Figure 15:**
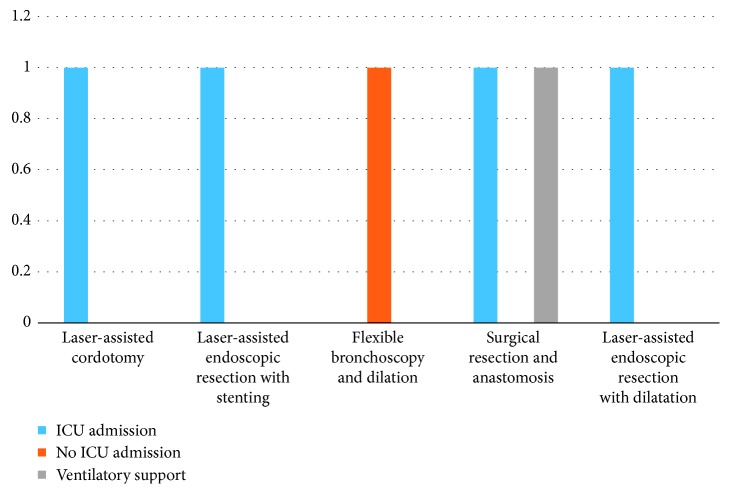
Patients requiring ICU/ventilatory support.

Postoperative intubation and ventilator support were required in 1 patient, and remaining 4 patients did not requir any assisted ventilation postoperatively (Figure 15). ICU admission was required for 4 patients out of 5. Median length of ICU stay was 5.5 (range: 4–7 days)

The median length of hospital stay was 9 days (range: 7–21 days). The postoperative success rate was 100%. None of the 5 patients in the study had severe perichondritis and postoperative stridor.

### 3.4. Follow-Up Review

The median follow-up duration was 6 months (range: 2–12 months). All the 5 patients were asymptomatic and very satisfied with their results after the procedure. The patient who underwent resection and anastomosis by open surgical technique and using holmium laser with balloon dilatation had improvement in phonation immediately after 1-month follow-up. The patient with bilateral vocal cord paralysis had no airway problems, and the patient with laser-assisted resection with stenting and phonation improved over the six-month follow-up period. The patients who underwent flexible bronchoscopy with dilation alone had no airway complaints, but there was not much improvement in phonation.

## 4. Discussion

Endotracheal intubation is a known cause of glottic/subglottic stenosis. The other important causes being complication of tracheostomy and surgical/external trauma.

Postintubation stenosis is a clinical problem caused by regional ischemic pressure necrosis of the airway [7]. Stenosis can occur anywhere from the level of the endotracheal tube tip till the glottic and subglottic area. The most common sites of stenosis are where the endotracheal cuff has been in contact with the tracheal wall and at the tracheal stoma site following a tracheostomy [8]. As a result, stenosis occurs most commonly following the two types of airway intubation: endotracheal intubation and tracheostomy [9].

Causative factor of stenosis is the pressure exerted by the cuff on the tracheal mucosa. At a cuff pressure of >30 mmHg, there is an increase in mucosal capillary perfusion pressure, which leads to mucosal ischemia and consequent inflammation of the tracheal cartilages. These pathological changes may eventually lead to fibrosis of circumferential lesions, resulting in progressive tracheal stenosis [10]. Ischemic injury can occur even within minutes after insufflation of the cuff and subsequent fibrotic changes within the following 3–6 weeks. Although the use of large-volume, low-pressure cuffs markedly reduces the occurrence of cuff injury, glottic stenosis continues to occur at a high frequency, with the incidence of postintubation tracheal stenosis in intensive care units being 6–21%, although only 1-2% of patients are symptomatic or have severe stenosis [3].

The diagnosis of laryngotracheal stenosis is often missed, and the related symptoms are generally evident only when stenosis of 30% of the original diameter of the trachea has occurred. Sometimes, the diagnosis may be delayed for as long as three months after the intubation [4]. Several risk factors of postintubation stenosis have been recognized thus far, including the size of the endotracheal tube relative to the tracheal lumen, frequent replacement of the endotracheal tube, traumatic intubation, concurrent infection, blood pressure during the intubation period, female gender, estrogen effect, steroid administration, obesity, and smoking history [5, 6].

Intubation needed during treatment is the most likely cause of stenosis in the three cases. Development of stenosis following intubation has been reported to occur even with two days of intubation [9]. A period up to two weeks in adults and even longer in children is generally considered safe.

In the above 5 cases, patients were asymptomatic after discharge from their primary care and started showing symptoms of stenosis within 1 month.

The factors that are related to development of stenosis with shorter duration of intubations are large size of the tube, high pressure in cuff, not deflating cuff periodically, struggling or restless patient, traumatic intubation, multiple intubation, and infection around the cuff site [9, 11, 12]. All these factors may play a role in development of stenosis.

The assessment of the degree of stenosis is an important step for each patient. Although several grading systems have been proposed, the universally accepted grading of subglottic stenosis was devised by Myer et al.: grade 1, <50% obstruction; grade 2, 51–70% obstruction; grade 3, 71–99% obstruction; and grade 4, no detectable lumen [13].

Galluccio et al. proposed classifying subglottic stenosis as either simple or complex [14]. They defined simple stenosis as lesions <1 cm in length with no associated tracheomalacia or loss of cartilaginous support. Complex lesions were >1 cm and had the greatest benefit from surgical intervention. This is significant for our study as our patients had complex lesions.

Complications of open surgical end-to-end anastomosis include restenosis, dehiscence, granulation, dysphagia, and RLN damage.

Granulation tissue formation occurs in proportion to the traction at the anastomosis site; choosing an appropriate type of suture is important, and we preferred polypropylene. Suture tension should be appropriate, and the suture knot should be formed outside the trachea to prevent formation of granulation tissue. Behrend and Klempnauer used three types of suture material (polypropylene, polydioxanone, and polyglactin) in tracheal surgery in sheep [15]. The results were similar in all three groups, but it was noted that the suture material should be of high tensile strength and should not be absorbed in under six months. The authors concluded that the technical details (especially tension) are more important than the choice of suture material for postoperative results.

In case 1, the patient had previous history of 4 endoscopic dilations followed by stenting, and case 2 underwent dilation prior to laser excision; both patients were not relieved of their complaints. In case 4, the patient had undergone plain flexible bronchoscopy with dilation, and there was no evidence of recurrence postoperatively. Dilation is achieved with lubricated bougies of increasing diameter applying radial pressure circumferentially to the narrowed airway. Balloon dilation is an alternative method. Rigid bronchoscope can be used to perform blunt dissection and dilation of stenosed areas under direct vision.

For all dilating techniques, it is important that the path of the true airway lumen is identified [16]. Preoperative imaging is useful for defining patient anatomy. With the associated risk of perforation and the high chance of recurrence of stenosis, one can see that dilation alone is very rarely a definitive therapy (especially in complex, high-grade stenosis) and patients will ultimately need surgical intervention.

There is a paper published in 2014 by Ortiz et al., who treated 18 children with repeated endoscopic tracheal dilation [17]. There was no recurrence of stenosis in any of the patients (median follow-up duration: 36 months, range: 5–72 months). However, this finding may not be replicable in the adults. Children have more favourable results with tracheal dilation as their inflammatory response is less pronounced, and there is therefore less fibrous tissue formation, meaning they have a lower risk of restenosis. Ortiz et al. [17] applied mitomycin C as an antifibroblast agent to reduce the chance of recurrence. In case 5, mitomycin C was applied after laser excision and balloon dilatation. There was no evidence of recurrence after 6-month follow-up.

There is a lot of literature supporting the use of stents in treating benign and malignant laryngeal stenosis. Dass et al. [18] conducted a study between the year 2000 and 2010, where out of 111 patients with laryngotracheal trauma, 71 underwent tracheal T-stenting for laryngotracheal stenosis. It was concluded that the ideal treatment option should be individualized based on patient characteristics. Stenting remained a relatively conservative treatment, was successful in a proportion of cases, and does not preclude the possibility of future reconstructive surgery if it fails. In case 1, our patient who subsequently underwent open resection and anastomosis of the stenotic segment had history of stenting after multiple failed dilations which provided him with little improvement. In case 2, the patient who underwent laser-assisted resection was discharged with a stent, and there was no evidence of recurrence.

Another method of treatment for postintubation laryngotracheal stenosis is neodymium-doped yttrium aluminium garnet laser and cryotherapy. They have not been reported as being used in the treatment of postintubation laryngeal stenosis except in very small series or case reports with no long-term follow-up review.

In two of our cases (case 2 and 3), CO_2_ diode laser was used for excision of the subglottic stenosis. In one of our cases (case 5), holmium laser was used for subglottic stenosis excision. In all the three cases of laser, there was improvement in airway and no evidence of stridor postoperatively.

It is seen that open surgical procedures involved certain potential risks not associated with endoscopic techniques including increased anaesthesia time and lengthy hospitalization. Because of these reasons, there has been a general tendency to treat laryngotracheal stenosis with endoscopic techniques. Addition of laser has offered new dimension in improving the conventional endoscopic technique in the management of laryngotracheal stenosis. Use of laser coupled to a rigid/flexible bronchoscope and ventilating bronchoscope allowed the surgeon to perform hands off endoscopic surgery such as scar excision or vaporization with increased precision. In our three cases treated by endoscopic laser, the patient could be decannulated after the procedure giving a fairly good success rate. Simpson et al. [19] successfully treated 1 of 5 (20%) patients having combined laryngeal and tracheal stenosis with endoscopic laser excision. Minimal pain, low levels of intraoperative and postoperative oedema, faster healing with less scar, and improved hemostasis are advantages of laser use.

There is a reduced risk of inadvertent injury to distal structures. A major advantage of the holmium laser is that it is capable of delivering energy to the target tissue through air, saline, or blood. Thus, it is particularly effective in wet environments such as the tracheobronchial tree. The ease of use with a fiber also enables this laser to be used endoscopically with flexible scopes. Hence, in case 5, holmium laser was preferred [20].

There is much evidence in the literature suggesting that for the treatment of subglottic stenosis, resection by endoscopy with laser and open surgical technique end-to-end anastomosis are the best available modality with regard to long-term results [21–24].

Our study supports this theory to perform resection for stenosis by end-to-end anastomosis or endoscopy with laser on patients who have had previous interventions.

## 5. Conclusion

Endotracheal intubation is life-saving when there is a need for artificial ventilation, but it is not without risk. Development of tracheal stenosis, that too of higher grade, is one of the most dreaded complications. However, such conditions can be managed with high degree of success.

Resection and primary anastomosis and laser-assisted excision of stenotic portion is a safe option for the treatment of subglottic stenosis following intubation in grade 3 or 4 stenosis without the need for repeated dilation. Collaboration is needed between thoracic and ENT surgeons to develop protocols for management of postintubation laryngotracheal stenosis as patients may present to both specialties.

Improved hemostasis, lesser pain, lesser postoperative oedema, and faster healing with less scar are advantages of laser, thus making it a safe option in management of tracheal stenosis.

Postoperative improvement in phonation was seen in surgical resection anastomosis, laser-assisted cordotomy, laser-assisted excision with dilation with mitomycin C application, and laser excision with stenting as compared to flexible bronchoscopy with dilation alone.

Given the high rates of recurrence, we cannot see a role for dilation alone in centres where specialised tracheal surgeons are available. Endoscopic dilation should be reserved for unfit patients or as a measure in nonequipped centres.

## Figures and Tables

**Table 1:** Reason for tracheostomy/intubation.

	Cases (*n*=5)
Poisoning	4 (80%)
Head injury	1 (20%)

**Table 2:** Comorbidities.

	Cases (*n*=5)
Male/female	4/1
Age (median range)	22 ± 10 years (range: 13 to 33 years)
Diabetes mellitus	1 (20%)
Cardiovascular disease	0
Systemic hypertension	0
Gastroesophageal reflux disease	0
Chronic obstructive pulmonary disease	0
Asthma	0
Obstructive sleep apnea	0
Hypothyroidism	1 (20%)
Alcohol	2 (40%)
Smoking	2 (40%)
Corticosteroid therapy	3 (10%)

**Table 3:** Characteristics of stenosis.

	Cases (*n*=5)
Days with endotracheal tube	6.8 (7–10)
Days with tracheostomy	206.25 (14–900)
Distance from vocal cords (cm)	3.1 (2–7)
Distance from main carina (cm)	3.6 (3–5)
Length of stenosis (cm)	1.2 (0.5–2.5)
Percent of stenosis	78.5% (60–90%)

**Table 4:** Treatment modalities.

	Patients (*n*=5)
Flexible bronchoscopy with dilation	1
Open surgical anastomosis	1
CO_2_ laser-assisted cordotomy with removal of subglottic stenosis by laser	1
CO_2_ laser-assisted resection with Montgomery tube stenting	1
Holmium laser-assisted resection of stenosis with balloon dilatation with application of mitomycin C	1

## References

[1] Grillo H. C., Donahue D. M., Mathisen D. J., Wain J. C., Wright C. D. (1995). Postintubation tracheal stenosis. Treatment and results. *Journal of Thoracic and Cardiovascular Surgery*.

[2] Anand V. K., Alemar G., Warren E. T. (1992). Surgical considerations in tracheal stenosis. *The Laryngoscope*.

[3] Dutau H. Tracheal stenosis endoscopic treatment.

[4] Papla B., Dyduch G., Frasik W., Olechnowica H. (2003). Post-intubation tracheal stenosis-morphological-clinical investigations. *Polish Journal of Pathology*.

[5] Esteller-Moré E., Ibañez J., Matiñó E., Ademà J. M., Nolla M., Quer I. M. (2005). Prognostic factors in laryngotracheal injury following intubation and/or tracheotomy in ICU patients. *European Archives of Oto-Rhino-Laryngology*.

[6] Vandemoortele T., Laroumagne S., Bylicki O., Astoul P., Dutau H. (2013). Endobronchial treatment of complete tracheal stenosis: report of 3 cases and description of an innovative technique. *Annals of Thoracic Surgery*.

[7] Wain J. C. (2003). Post intubation tracheal stenosis. *Chest Surgery Clinics of North America*.

[8] Nouraei S. A., Ma E., Patel A., Howard D. J., Sandhu G. S. (2007). Estimating the population incidence of adult post-intubation laryngotracheal stenosis. *Clinical Otolaryngology*.

[9] Zias N., Chroneou A., Tabba M. K. (2008). Post tracheostomy and post intubation tracheal stenosis: report of 31 cases and review of the literature. *BMC Pulmonary Medicine*.

[10] Spittle N., McCluskey A. (2000). Tracheal stenosis after intubation. *BMJ*.

[11] Cinnamond M. J., Kerr A. G., Groves J., Evans J. G. (1987). Stridor. *Scott-Brown’s Otolaryngology*.

[12] Keshava K., Weingarten J. A., Grosu H. B. (2013). “Benign” tracheal stenosis in an 18-year-old man. *Annals of the American Thoracic Society*.

[13] Myer C. M., O’Connor D. M., Cotton R. T. (1994). Proposed grading system for subglottic stenosis based on endotracheal tube sizes. *Annals of Otology, Rhinology & Laryngology*.

[14] Galluccio G., Lucantoni G., Battistoni P. (2009). Interventional endoscopy in the management of benign tracheal stenoses: definitive treatment at long-term follow-up. *European Journal of Cardio-Thoracic Surgery*.

[15] Behrend M., Klempnauer J. (2001). Influence of suture material and technique on end-to-end reconstruction in tracheal surgery: an experimental study in sheep. *European Surgical Research*.

[16] Bacon J. L., Patterson C. M., Madden B. P. (2014). Indications and interventional options for non-resectable tracheal stenosis. *Journal of Thoracic Disease*.

[17] Ortiz R., Dominguez E., Torre C. (2014). Early endoscopic dilation and mitomycin application in the treatment of acquired tracheal stenosis. *European Journal of Pediatric Surgery*.

[18] Dass A., Nagarkar N. M., Singhal S. K., Verma H. (2014). Tracheal T-tube stent for laryngotracheal stenosis: ten year experience. *Iranian Journal of Otorhinolaryngology*.

[19] Simpson G. T., Strong M. S., Shapsay S. M., Healy G. B., Vaugham C. W. (1982). Predictive factors of success or failure in the endoscopic management of laryngeal and tracheal stenosis. *Annals of Otology, Rhinology & Laryngology*.

[20] Verret D. J., Jategaonkar A., Helman S. (2017). Holmium laser for endoscopic treatment of benign tracheal stenosis. *International Archives of Otorhinolaryngology*.

[21] George M., Lang F., Pasche P., Monnier P. (2005). Surgical management of laryngotracheal stenosis in adults. *European Archives of Oto-Rhino-Laryngology*.

[22] Marques P., Leal L., Spratley J., Cardoso E., Santos M. (2009). Tracheal resection with primary anastomosis: 10 years experience. *American Journal of Otolaryngology*.

[23] Grillo H. C., Donahue D. M., Mathisen D. J., Wain J. C., Wright C. D. (1995). Postintubation tracheal stenosis. *Journal of Thoracic and Cardiovascular Surgery*.

[24] Hassan F. H., Goh B. S., Kong M. H., Marina M. B., Sani A. (2013). Tracheal resection and anastomosis: an 11 year management outcome. *Rawal Medical Journal*.

